# Food for Infants during Dentition

**Published:** 1875-08

**Authors:** B. F. Dawson


### ARTICLE V.

### *Food for Infants During Dentition.*

BY DR. B. F. DAWSON.

There is a prevailing opinion that the child requires a much more substantial food when the teeth begin to appear, and also that the existence of a diarrhœa of greater or less severity, during this period, is a thing to be expected; consequently, when the child suffers from any gastro-intestinal trouble while the teeth are cutting, it is altogether dependent upon that process. The doctor expressed his conviction that faulty alimentation is the chief cause in the production of the gastro-intestinal troubles met with during this period, and that if the child requires a more substantial diet, it should be only such as contains milk as its chief base.

The paper was listened to with great attention, and the author concluded by saying that not all gastro-intestinal diseases could be avoided with the most assiduous care and attention to the diet of children; but that, as a result of his experience, he believed a great majority of these cases are due to improper alimentation, both as regards the quantity and quality of the food administered.

Dr. Joel Foster remarked that he regarded the paper of the evening as plain and practical. He had been particularly interested with regard to the question of over-feeding infants, and believed that almost as much mischief can be done in this way as by under-feeding. He was also convinced of the very great necessity of the mother nursing the child during the earlier period of its life, for she alone can furnish the real natural food for it.

With regard to artificial food, it was a subject in which he was particularly interested as being connected with the New York Infant Asylum. In that institution it is impossible in many cases to get natural food, and they are obliged to use some substitute, and the substitute which can best be made is cow's milk. The method of preparing it which he had followed was to allow the milk to stand until the cream begins to rise, and then take the upper portion and dilute it with barley-water. He was then particular that the food should be given at regular intervals, and it should always be so given at a temperature near that of the human body, for if given below that degree it is very likely to produce gastro-intestinal troubles, which destroy so many children. It had been found that milk taken directly from the cow and given to the children did not do nearly as well as when permitted to stand for about two hours, when a partial separation of the cream has taken place, and then taking the upper portion of the milk. In this way more fat and less casein is obtained.

Dr. Porter held that perhaps hot weather and other influences had very much to do with producing this class of diseases as well as improper alimentation.

Dr. Messinger urged the propriety of thoroughly cooking whatever article was used for diluting the milk, and he always insisted that the barley-water should be boiled for three or four hours.

Dr. Garrish objected to keeping the milk upon which the child is to be fed in the ordinary ice-box which contains meats, vegetables, etc. A convenient refrigerator can be made of a common stone crock, by placing a piece of ice wrapped in flannel in the bottom of it, and there the milk can be kept with the greatest ease.

Dr. McIlvaine regarded dilution of cow's milk as improper. He believed that the parotid glands secrete from the earliest period of life, and looked with a great degree of caution upon the statements made concerning experiments said to prove to the contrary.

Dr. Roberts referred to one point which had not been spoken of with regard to cow's milk, and that was that it is generally acid, while mother's milk will usually be found to be alkaline. He did not think that cow's milk is the best substitute that can be used for breast milk.

Dr. Hanks spoke with reference to the fact that among that class of people where gastro-intestinal diseases prevail most extensively, the children are poorly cared for as well as ill fed, and that it is a very common thing to find them overburthened with clothing during the hot weather.

Dr. Lewis Smith remarked, with reference to the use of farinaceous food, that up to the third month the salivary glands and pancreas are present only in a rudimentary state consequently that fluid which is afforded by the animal economy for the digestion of starchy material is absent, but it is also probably true that the starch is not so irritating as is casein undigested. He has been accustomed to use the upper portion of the milk after it has stood for a short time, and prefers to use some article for dilution in which the starch has been changed into dextrine or glucose, and recommends Liebig's food. He does not think that sugar should be added at all during warm weather when diarrhœa is present. If there is constipation he gives sugar, and sugar of milk is the best form in which it can be employed. He was of the decided opinion that many deaths occur among children from the fact that the mother regards eight, ten or twelve stools every day as necessary while the child is teething. This widespread error should, if possible, be corrected.

Dr. Robinson suggested that the weight of the child might determine whether it was receiving sufficient food or not. Decrease in weight might be due to other causes, but a careful observation would enable us to determine whether the decrease was due to insufficient supply of food or not. —*N. Y. Medical Library and Journal Association, in Medical Record.*

---

